# Prevalence of cognitive impairment and its predictors among chronic kidney disease patients: A systematic review and meta-analysis

**DOI:** 10.1371/journal.pone.0304762

**Published:** 2024-06-03

**Authors:** Jialing Zhang, Leiyun Wu, Peixin Wang, Yajing Pan, Xingtong Dong, Linpei Jia, Aihua Zhang

**Affiliations:** 1 Department of Nephrology, Xuanwu Hospital, Capital Medical University, Beijing, China; 2 The National Clinical Research Center for Geriatric Disease, Xuanwu Hospital, Capital Medical University, Beijing, China; Baghdad Medical City, IRAQ

## Abstract

**Background:**

Cognitive impairment (CI) is common among patients with chronic kidney disease (CKD), and is associated with a poor prognosis. We assessed the prevalence and associated factors of CI in patients with CKD.

**Methods:**

A systematic review and meta-analysis were conducted by searching PubMed, Embase, and the Web of Science through December 1, 2023. Random effects models were performed with subgroup analyses to further explore the heterogeneity.

**Results:**

50 studies involving 25,289 CKD patients were included. The overall prevalence of CI was 40% (95% confidence interval 33–46). The pooled prevalence of CI was relatively higher in CKD patients from Africa (58%), Asia (44%) and America (37%). Attention and executive dysfunction appeared to be the most common manifestations. The prevalence of CI was higher among patients with hemodialysis (53%) and peritoneal dialysis (39%) than those without dialysis (32%) and post-kidney transplanted (26%). In addition, advanced age, the presence of diabetes and hypertension might increase the risk of CI in CKD patients.

**Conclusions:**

People with CKD have a high prevalence of CI, especially in patients with hemodialysis. An early and comprehensive screening for CI in CKD patients is needed to improve clinical outcomes.

**Trial registration:**

**Registration number:** PROSPERO (CRD42023412864)

## Introduction

Chronic kidney disease (CKD) is one of the most common causes of morbidity and mortality worldwide [1]. CKD is identified based on the degree of kidney damage or by a measured glomerular filtration rate (GFR) ≤60 mL/min/1.73 m^2^ for more than 3 months [2]. The global prevalence of CKD was estimated to be 13.4% [3]. Cognition impairment (CI) in patients with CKD has become a major concern. It is well established that CI may positively correlate with the degree of decline in residual renal function [4], even in early CKD [5]. A decrease in multiple cognitive functions, including processing, memory, and executive function, has been shown in CKD patients [6]. Moreover, magnetic resonance imaging techniques showed that hippocampus atrophy, cortical atrophy, and white matter lesions are frequent in CKD patients, and are likely to lead to cognitive disturbance [7, 8].

CI was independently associated with mortality in elderly hemodialysis (HD) patients [9]. However, it should be noted that the prevalence of CI in patients with CKD are variable. Kurella et al. reported a 23–28% prevalence of CI in stage 3–4 CKD patients [10], nearly 87% in patients with HD [11], and 50% in patients with peritoneal dialysis (PD) [12]. The prevalence of CI in CKD patients varies widely around the world, reaching 58% in the USA [13], 49.9% in Asia [12], 30.3% in Europe [14], and 35% in Africa [15]. However, quantitative studies on the current epidemiology of CI in patients with CKD are few.

The pathophysiology of cognitive decline in CKD patients is complex. Various clinical factors could contribute to CI in CKD, including traditional risk factors (e.g., older age, education level, diabetes, and dyslipidemia) and other specific factors (e.g., dialysis modality, anemia, vitamin D) [16, 17]. It is imperative that the risk factors and prompt treatment of CI in the CKD population should be emphasized.

Thus far, studies regarding the prevalence of CI in CKD patients are limited and with inconsistent conclusions. Our study aimed to estimate the overall prevalence of CI in CKD patients not on dialysis, and receiving dialysis or kidney transplantation. Furthermore, the specific risk factors for CI were also explored.

## Materials and methods

### Protocol and registration

The protocol for this systemic review and meta-analysis was registered on PROSPERO (CRD42023412864). The reporting was done according to the Preferred Reporting Items for Systematic reviews and Meta-Analyses (PRISMA) Protocols guidelines [18].

### Search strategy and selection criteria

A search was conducted via PubMed, Embase, and Web of Science for relevant articles. The search was conducted from January 1, 2000 to December 1, 2023 with the Medical Subject Headings terms ‘chronic kidney failure’, ‘dialysis’, and ‘cognitive dysfunction’. We also used the free terms ‘chronic kidney disease’, ‘chronic kidney insufficiency’, ‘hemodialysis’, ‘cognitive impairment’, and ‘cognitive defect’. The search strategy was adjusted based on the compatibility of each database and shown in **S1 Checklist**. Additionally, the reference lists of the included studies were reviewed to identify other relevant studies.

Two investigators independently selected relevant studies based on the following inclusion criteria: (1) studies specified the primary data on the prevalence of CI in CKD patients; (2) study patients aged 18 years and above; (3) study patients with CKD or undergoing any kidney replacement therapy (HD, PD, and kidney transplant); (3) observational studies, including cross-sectional studies and cohort studies; and (4) included at least 50 participants. Articles without prevalence data, without the diagnosis criteria of CI, not written in English, or studies in the form of commentaries, editorials, reviews, or case reports were excluded. For overlapping data of the same cohorts, we included only the most comprehensive data.

### Data extraction and risk of bias assessment

Two authors (J.L.Z. and L.Y.W.) independently selected the articles by screening the title, abstract and full text according to eligibility criteria. The inconsistencies were resolved by a third author (A.H.Z.). Data extraction of eligible articles, including author, year of publication, study design, sample size, baseline characteristics of the patients, and measurement tools for CI, was independently carried out by two reviewers (J.L.Z. and L.Y.W.). The risk of bias was assessed using the Newcastle–Ottawa Scale (NOS) [19]. A total of <3 points was considered low quality, 4–6 points was considered moderate quality, and 7–10 points was considered high quality.

### Statistical analysis

Meta-analysis was conducted in Stata version 15 statistical software. Analyses of pooled estimates of the prevalence of CI in CKD patients were presented as percentages with a 95% confidence interval (95% CI) using forest plots. Heterogeneity was assessed for the statistical significance using I^2^ [20]. A random-effects model was employed when I^2^ >50%. Subgroup analysis was performed stratified by publication year, dialysis modality, regions, diagnostic instruments, and degree of CI to further investigate the heterogeneity across studies. Besides, the publication bias was evaluated by Egger’s regression test. In terms of associated factors for CI, the adjusted odds ratio (OR) with a corresponding 95% CI was calculated. A P value < 0.05 was considered to be statistically significant.

## Results

### Characteristics of the included studies

**Fig 1:**
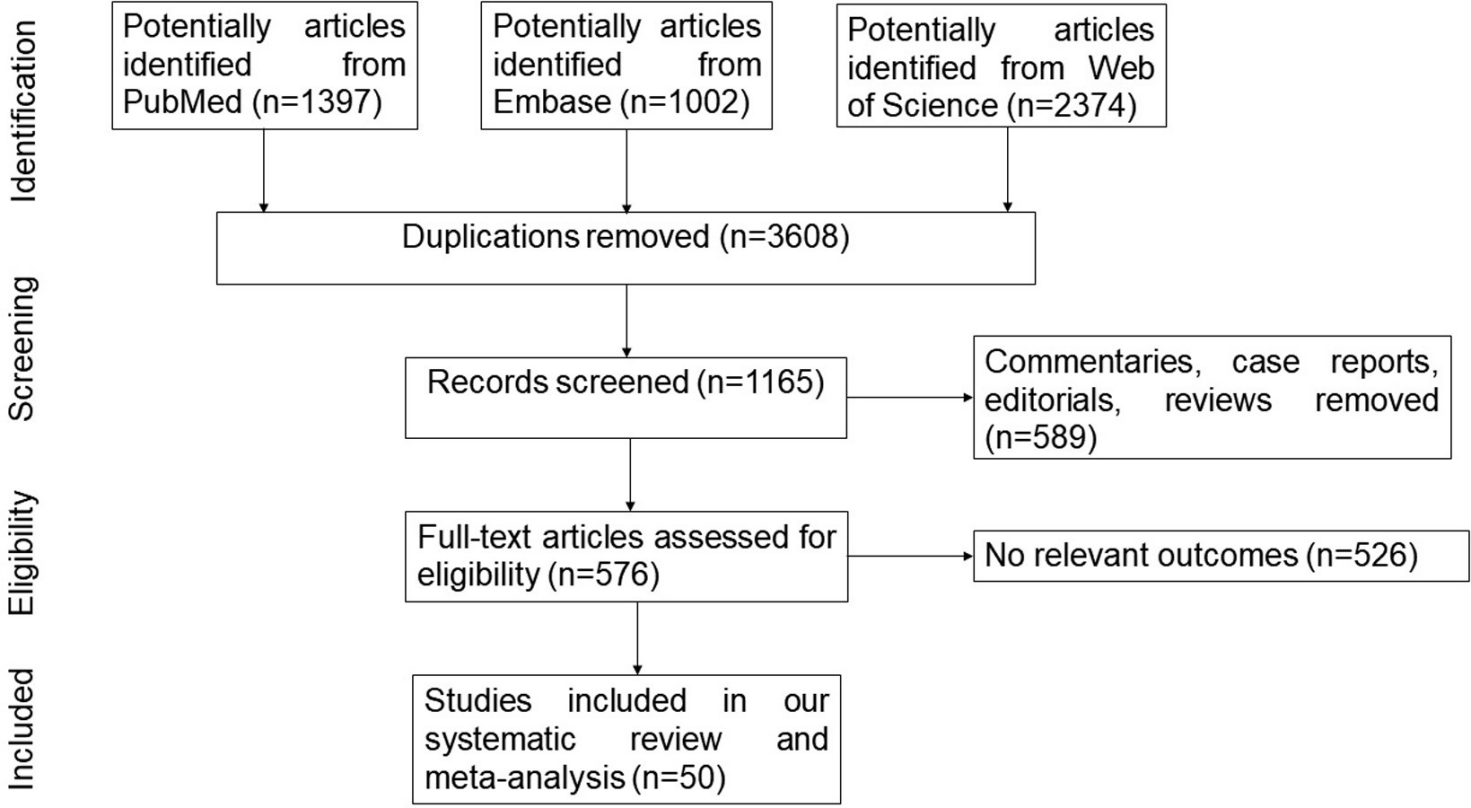
PRISMA flow diagram.

A total of 4773 publications were identified at the three electronic databases in the initial search, and 50 studies [12–15, 21–65] were included according to the selection criteria. **Fig 1** represented the flowchart of the selection of studies for our systematic review and meta-analysis. **S1 Table** summarized the characteristics of the selected articles. In total, 25,289 CKD patients were included across 50 studies. Among these studies, 16 studies were conducted in America, 16 were from Asia, 3 were across Africa, 14 were conducted in Europe, and 1 was from Australia. 12 studies involved CKD patients without dialysis, 17 studies involved patients on HD, 10 studies involved patients on PD, and 6 studies involved patients with kidney transplant. Multiple instruments have been used to evaluate cognitive function. 15 studies used Montreal Cognitive Assessment (MoCA), 25 studies used Mini-mental State Examination (MMSE), and 2 studies used Adden brooke’s Cognitive Examination III (ACE III).

### Prevalence of CI in patients with CKD

**Fig 2:**
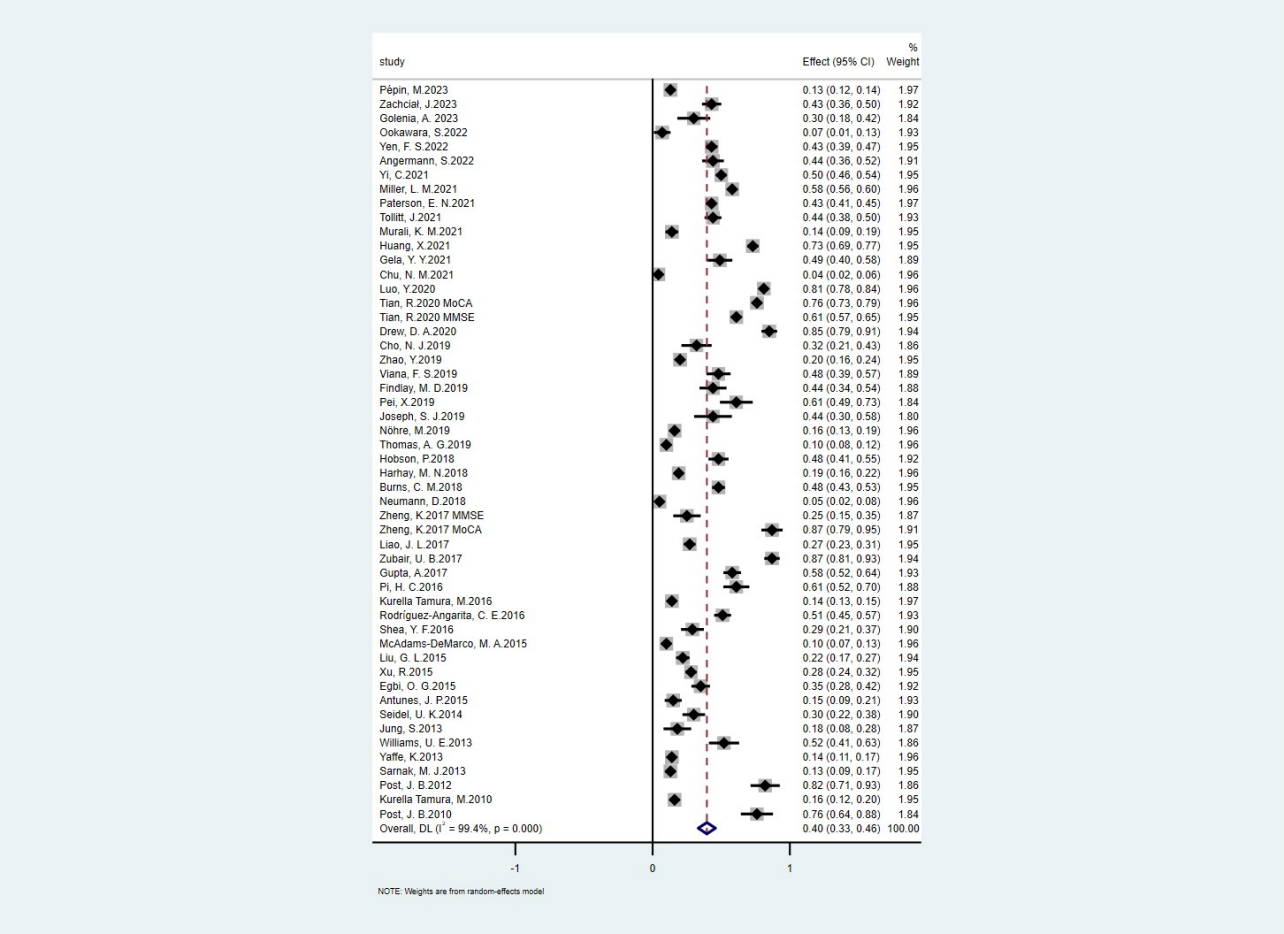
Forest plot of the pooled prevalence of cognitive impairment among CKD patients. NOTE: Weights are from random-effects model.

The results of the primary analyses were presented in **Fig 2**. The pooled prevalence of CI among CKD patients was estimated to be 40% (95% CI, 33–46) with a significant heterogeneity as evidenced by I^2^ statistic (I^2^ = 99%; p < 0.0001). Hence, a random effect model was employed considering the significant heterogeneity among the studies. Egger’s test provided evidence of publication bias in the overall meta‐analysis (P = 0.002).

### Subgroup analysis

**Table 1** summarized the subgroup analyses determining the pooled prevalence of CI in CKD patients. To pinpoint the source of heterogeneity, subgroup analyses were performed stratified by the study area, year of publication, type of cohorts, assessment tools, cognitive subdomains, and degree of CI. The forest plots were shown in **S1-S6 Figs in S1 File**. The prevalence of CI was 37% in USA, 58% in Africa, 44% in Asia, 34% in Europe, and 14% in Australia, respectively. Based on the patients’ type, the highest prevalence was identified in patients with HD (53%), followed by patients with PD (39%), CKD patients without dialysis (32%) and post-kidney transplanted patients (26%). Furthermore, the pooled prevalence of CI was 57% assessed by MoCA, 27% assessed by MMSE, and 40% assessed by ACE III. Among the various cognitive subdomains, 29% had impaired attention; 28% had impaired executive function; 18% had impaired delayed memory, and 16% had impaired language. According to the prespecified criteria from original studies, 35% of the patients had mild CI, and 33% had moderate-severe CI. In addition, we estimated the prevalence of CI according to year of publication. The prevalence of CI was higher after 2015 than before 2015 (42%, 95% CI 35%-50%, and 31%, 95% CI 23%-38%).

**Table 1:** The pooled prevalence of cognitive impairment in CKD patients by subgroup analysis.

	Prevalence (%)	95% confidence interval	I^2^ (%)
Country			
Africa	58	22–94	98.6
Australia	14	9–19	0
Asia	44	31–56	99.1
Europe	34	25–44	99.0
America	37	27–47	99.5
Cohort type			
Hemodialysis	53	38–69	99.3
Peritoneal dialysis	39	26–53	98.6
Non-dialysis	32	25–38	100.0
Kidney transplant	26	15–38	98.6
Assessment tool			
MoCA	57	49–64	97.1
MMSE	27	22–32	98.3
Addenbrooke’s Cognitive Examination III	40	22–57	84.1
Subdomain			
Attention	29	16–42	98.0
Executive function	28	20–36	96.4
Language	16	14–18	0
Memory	18	13–23	75.2
Degree of CI			
Mild	35	26–44	94.3
Major	33	20–46	97.8
Study year			
Below 2015	31	23–38	96.7
Over 2015	42	35–50	99.5

### Factors associated with CI among CKD patients

Six studies evaluated the association between the older age and the presence of CI among CKD patients. The pooled OR showed that older CKD patients were more likely to have CI (OR = 1.07, 95% CI:1.04–1.11). The analysis of four studies showed that prevalence of CI was comparable between male and female CKD patients (OR = 0.88, 95% CI:0.67–1.16). CKD patients with diabetes or hypertension were likely to have a significantly higher rate of CI (OR = 1.35, 95% CI:0.99–1.86; OR = 1.88, 95% CI:1.1–3.22). However, the status of smoke or drink did not affect the prevalence of CI in CKD patients (OR = 0.69, 95% CI:0.44–1.08; OR = 0.79, 95% CI:0.44–1.4) (S7 Fig in S1 File).

## Discussion

To our knowledge, this is the first systematic review and meta-analysis to assess the prevalence of CI in patients with CKD stage 1 to 5 and the population on HD, PD or kidney transplantation. We carried out a comprehensive systematic review and meta-analysis of 50 studies that included 25,289 CKD patients to investigate the prevalence of CI. The overall prevalence of CI was 40% in CKD patients. We observed high prevalence of CI (39% and 53%) in patients undergoing PD and HD in comparison to patients after kidney transplantation (26%). Overall, these data highlighted the burden of CI, and the importance of early detection and treatment for CI in CKD patients should be emphasized.

CKD is a growing health problem worldwide [66]. A decrease in cognitive function might be correlated with the severity of renal failure [67]. A cross-sectional study reported that each 10-mL/min/1.73 m^2^ decrease in the estimated GFR less than 60 mL/min/1.73 m^2^ was associated with an 11% increase in the prevalence of CI [68]. The pathophysiology of CI in CKD patients is multifactorial and undefined. Traditional cardiovascular risk factors and various kidney-disease related factors, including uremic toxins, dialysis modality, complications of dialysis, and anemia, have been identified [6]. The prevalence of CI varied widely in patients with CKD, ranging from 5% to 87% [35, 37]. On the basis of our meta-analysis, we reported the disparities in the prevalence of CI in CKD patients from different ethnic groups. The wide discrepancies in the burden of CI among CKD patients might be due to the different sample sizes, high heterogeneity of the study population characteristics, differences in the socioeconomic status, stage of CKD, access to diagnostic facilities and quality of healthcare services. Higher Medicare total annual cost of care significantly correlated with dementia [69]. Additionally, lifestyle, containing diets and physical activity, varied between countries and may potentially impact the prevalence of CI. Mediterranean diet and exercise could significantly lower the incident risk of cognitive disorders [70–72]. However, some other regions of the developing world were not represented in this analysis, probably leading to a variation. Additional studies exploring the prevalence of CI in CKD patients from developing countries are needed. An increasing trend in the global burden of CI in CKD patients was reported from the years below 2015 to the years over 2015. The differences might be explained by other comorbid conditions, unhealthy lifestyles, awareness of healthcare utilization and poor-quality healthcare services [73].

The effect of dialysis modalities on the cognitive functions was debated. The 5-year cumulative risk of dementia in PD patients was lower than that in HD patients in a large retrospective study [74]. In our study, we also found that PD patients seemed to have a significantly lower prevalence of CI than HD patients. Various comorbidities, such as diabetes, hypertension and dyslipidemia, could be common in end-stage renal disease patients with HD, and these comorbidities might increase the risk of CI [75]. Evidence underscored that CI was considerably prevalent among individuals with hypertension [76] and diabetes [77]. The intervention to maintain blood pressure and serum glucose might benefit for cognition [78–80]. For example, dapagliflozin and vildagliptin could exert beneficial effects on neuroprotection [81]. Angiotensin-converting enzyme inhibitor and/or angiotensin receptor blocker could decrease odds of CI through ameliorating albuminuria [82]. In our meta-analysis, we also found that diabetes and hypertension were independent risk factors for CI in CKD patients. Besides, the residual kidney function might be better preserved in PD patients than in HD patients [83]. Patients with HD are more likely to suffer from a large fluid and osmolar fluctuation than patients with PD, probably leading to a decreased cerebral perfusion, and cerebral ischemia. On the other hand, the choice of dialysis method might be affected by autonomy, economic status, the level of education, and preferences. Patients likely need a better baseline cognition status to complete PD training programs. Consequently, the choice of renal replacement therapy in the clinical should be comprehensively considered. Evidence showed that post-transplanted patients performed better on general cognitive status, information and motor speed, spatial reasoning, and verbal memory when compared with dialysis-dependent patients [84]. The prevalence of CI in post-transplanted patients was also lower than that in HD and PD patients in this study. The risk of CI might decrease with a better renal function. Meanwhile, the neurotoxicity of calcineurin inhibitor after transplantation could not be ignored, either [85].

A high prevalence of white matter disease was shown in PD patients [86], accompanied by a decline in cognitive function [87]. A study including 51 PD patients showed that the prevalence of CI was approximately 75%, which was higher than that of our study. However, the number of patients included was small, which might lead to uncertainty. Among those patients, approximately one-third had moderate impairment, and one-third had severe impairment [88]. According to the degree of CI, our sub-analysis results showed that the prevalence of mild CI and major CI was 35% and 33% in CKD patients, respectively. Patients with mild CI even showed declines in the ability to perform instrumental activities of daily living, such as telephone calls, preparing hot meals, remembering to take medication, remembering appointments, talking about recent events, and performing hobbies [89]. A further comprehensive evaluation should be taken into consideration as early as possible in an individual with mild or moderate CI.

Clinical screening for CI relies on the use of cognitive tests. Regarding different diagnostic criteria, our meta-analysis illustrated that the prevalence of CI in CKD patients was highest when assessed by MoCA, followed by MMSE and ACE III. A study presented that the MoCA had greater sensitivity and specificity for detecting mild CI than does the MMSE [90]. Another small study of prevalent HD patients also revealed that the MoCA performed better than MMSE on neurocognitive tests [91, 92]. The pattern of CI in CKD is unclear. A previous meta-analysis showed that CKD patients performed worse on both attention, language, executive function, and memory [5]. However, cognitive performance in patients with dialysis and kidney transplant was not involved, and the robust prevalence data was absent. Subgroup analysis of this study indicated that the prevalence of attention dysfunction (29%) and executive dysfunction (28%) was higher than memory dysfunction (18%) and language dysfunction (16%). CI is common in patients treated with HD, particularly in the domains of attention and executive function [93]. The impairment of cognitive pattern seems to be different in PD patients, with more memory impairment [88]. Future research may propose more representative cognitive tests for multiple cognitive patterns in CKD patients.

Our current meta-analysis included a larger number of studies and more convincing articles to determine the prevalence of CI in CKD patients, likely leading to a more reliable conclusion. Our results recommended physicians to early diagnostics and intervene the progression of CI. Our study had several limitations. First, the heterogeneity was high in the meta-analysis of the prevalence of CI. Although several subgroup analyses were performed, the source of heterogeneity was not well defined. Second, the risk factors for CI extracted from the original studies were limited. Further studies are needed to determine the associated factors on CI in CKD patients, such as race, depression, nutritional patterns, and other dialysis-related factors. Additionally, the reports on efficacy and safety of antidementia drugs for CKD patients was too few to include in this meta-analysis. Third, the definition of CI was inconclusive across each individual study, which may lead to a bias. Fourth, the articles included were restricted only to those written in the English language.

## Conclusion

CI is highly prevalent in CKD patients worldwide, especially in USA, Africa and Asia. Our findings highlighted that the estimated prevalence of CI in CKD patients varied among different subdomains and degrees of CI. A screening for CI is urgently needed in CKD patients, particularly in patients undergoing HD and PD.

## Supporting information

S1 Checklist PRISMA checklist. (DOC)

S1 Table Characteristics of the included studies. (DOCX)

S1 File (DOCX)
