# Supplementary material for: Prevalence of cognitive impairment and its predictors among chronic kidney disease patients: A systematic review and meta-analysis
Source: PLoS One. 2024 Jun 3;19(6):e0304762. doi: 10.1371/journal.pone.0304762 (PMC11146742; doi:10.1371/journal.pone.0304762)
Supplement: S1 Table — (DOCX) [file pone.0304762.s002.docx]

**Table 1** Characteristics of the included studies

| Study | Country | Study design | Cohort | Sample size | Age (year) | Male (%) | DM (%) | HT (%) | CVD (%) | Duration of dialysis | Assessment for CI | NOS |
| --- | --- | --- | --- | --- | --- | --- | --- | --- | --- | --- | --- | --- |
| Pépin, M.2023 | France | CKD-REIN | CKD 3-4 | 3003 | 66.8±12.9 | 1961 (65.3) | 1292 (43.0) | 2718 (90.5) |  |  | MMSE | 8 |
| Golenia, A. 2023 | Poland | observational | KT | 56 | 50.3 ± 11.7 | 34 (61) |  |  |  | 26.7 ± 23.4 m | ACE III test | 7 |
| Zachciał, J.2023 | Poland | cross-sectional | KT | 190 | 61.7±12.1 | 101 (53.2) | 45 (23.7) | 153 (80.5) |  |  | MMSE | 8 |
| Ookawara, S.2022 | Japan | observational | CKD non-dialysis | 68 | 68.8±11.6 | 49 (72) | 27 (40) |  | 6 (9) |  | Modified MMSE | 7 |
| Yen, F. S.2022 | China | NHANES | CKD | 520 |  | 329 (63) | 173 (33) | 340 (65) |  |  | DSST | 7 |
| Angermann, S.2022 | German | observational | HD | 152 | non-CI 58.9±15.3; CI 70.8±11.5 | 106 (70) | 48 (32) |  |  | non-CI 70.3±69.1 m;  CI 55.3±56.2 m | MoCA | 7 |
| Yi, C.2021 | China | cross-sectional | PD | 643 | 45 (37–57.4) | 372 (58) | 105 (16) |  | 94 (15) | 27.8 (8.7–56.4) m | MoCA | 7 |
| Miller, L. M.2021 | USA | SPRINT | eGFR<60 mL/min/1.73 m^2^ | 2253 |  |  |  |  |  |  | MoCA | 7 |
| Paterson, E. N.2021 | UK | NICOLA | CKD | 3412 | 64±9 | 1604 (47) | 307 (9) |  |  |  | MoCA and MMSE | 7 |
| Tollitt, J.2021 | UK | prospective | CKD non-dialysis | 250 | 66 (53–74) | 164 (66) | 60 (24) | 225 (90.4) | 57 (22.8) |  | MoCA | 8 |
| Murali, K. M.2021 | Australia |  | ESKD | 211 | 66.9 ± 11.2 | 129 (61) | 91 (43.10) | 191(90.40) |  |  | MMSE | 7 |
| Huang, X.2021 | China |  | PD | 455 | 58.8±10.8 | 303 (66.6) | 418 (91.9) | 847 (92.8) | 161 (35.4) | 29.1 (6.5–54.2) m | MoCA | 8 |
| Gela, Y. Y.2021 | Ethiopia | cross-sectional | CKD | 116 | 54.1±17 | 75 (64.7) |  |  |  |  | MMSE | 7 |
| Chu, N. M.2021 | USA | cohort study | KT | 405 |  | 230(56.8) | 120 (29.8) |  |  | 2.4 (0.5, 5.5) y | Modified MMSE | 8 |
| Luo, Y.2020 | China | observational | HD | 613 | 63.8±7.1 | 355 (57.9) | 231 (37.7) | 545 (88.9) | 193 (31.5) | 57 (24, 101.5) m | Chinese Beijing version of the MoCA | 8 |
| Drew, D. A.2020 | USA |  | HD | 150 | 64±14 | 91 (61) | 76 (51) | 27 (18) |  | 2.7 (1.3, 5.6) y | Wechsler scale | 7 |
| Tian, R.2020 | China |  | HD | 613 | 63.7±7.8 | 355 (57.9) | 231 (37.7) | 545 (88.9) | 193 (31.5) | 57.0  (24.0-101.5) m | MoCA and MMSE | 7 |
| Cho, N. J.2019 | Korea | observational | HD | 69 | 55.3±11.4 | 37 (53.6) | 24 (34.8) |  | 10 (14.5) | 73.0  (37.0–125.0) m | Korean version of the MoCA | 8 |
| Zhao, Y.2019 | China | prospective | PD | 458 | 51.6±14.2 | 243 (53.1) | 108 (23.6) |  | 96 (21.0) | 25.1 (11.1–49.0) m | Modified MMSE | 7 |
| Findlay, M. D.2019 | UK | prospective | HD | 97 | 58 (50.5–66.5) | 62 (63.9) | 29 (33.0) | 78 (88.6) |  | 1.8 (0.6–4.0) y | MoCA | 7 |
| Viana, F. S.2019 | UK | cross-sectional | HD | 124 | 76.0 ± 6.2 | 69 (55.6) | 70 (56.5) | 121 (97.6) |  | 25 (11-58) m | MMSE | 7 |
| Joseph, S. J.2019 | India | cross-sectional | HD | 50 | 50.32±12.4 | 33(66) |  |  |  | 18.8±15.11 m | MoCA | 7 |
| Pei, X.2019 | China | cross-sectional | HD | 64 | 61±17 | 44 (68.8) | 16 (25.0) | 45 (70.3) |  | 2.9±2.2 y | MoCA | 7 |
| Nöhre, M.2019 | Germany | cross-sectional | KT | 583 | 52.1±14.3 | 344 (59) | 101 (17.4) | 501 (85.9) |  | 60.3±49.6 m | DemTect | 8 |
| Thomas, A. G.2019 | USA | prospective | KT | 864 | 53 (42–63) | 523 (60.6) | 150 (17.4) | 257 (29.8) |  | 1.9 (0.2–4.2) y | Modified MMSE | 8 |
| Hobson, P.2018 | UK |  | eGFR 15-60 mL/min/1.73 m^2^ | 178 | 76.1±8.2 | 97 (54.5) |  | 161 (90) |  |  | ACE III | 7 |
| Harhay, M. N.2018 | USA | CRIC | non-dialysis CKD (eGFR ≤ 20 mL/min/1.73 m^2^) | 630 |  | 284 (45.1) | 429 (68.1) |  | 304 (48.3) |  | Modified MMSE | 7 |
| Burns, C. M.2018 | USA | BRINK | non-dialysis CKD (eGFR ≤ 60 mL/min/1.73 m^2^) | 433 |  | 226 (52.2) | 228 (52.7) |  | 416 (96.1) |  | Modified MMSE | 7 |
| Neumann, D.2018 | German | CORETH project | PD+HD | 271 | 56.6±14.9 | 189 (69.7) |  |  |  | 14.8±5.3 m | 3-item subscale | 7 |
| Zheng, K.2017 | China | cross-sectional | PD | 72 | 56.2 ± 16.0 | 27 (37.5) | 23 (31.9) | 65 (90.3) |  | 41.2 ± 36.1 m | Chinese version of the MMSE and MoCA | 7 |
| Liao, J. L.2017 | China | cross-sectional | PD | 424 | 52.7±4.3 | 217 (51.2) | 117 (27.60) |  | 93 (21.9) | 33.2±27.5 m | MMSE | 8 |
| Zubair, U. B.2017 | Islamabad | cross-sectional | HD | 137 |  | 99 (72.3) |  |  |  |  | British Columbia Cognitive Complaints Inventory | 7 |
| Gupta, A.2017 | USA | cross-sectional | KT | 226 | 54±13.4 | 137(60.6) | 84 (37.2) |  | 42 (21.2) | 2.3±2.1 y | MoCA | 7 |
|  |  |  |  |  |  |  |  |  |  |  |  |  |
| Pi, H. C.2016 | UK | prospective | PD+HD+ CKD | 102 | HD 68.9 ± 1.3  PD 72.8 ± 1.6 CKD 72.5 ± 1.5 | HD 29 (70.7)  PD 19 (76.0); CKD 23 (63.9) | HD 19 (46.3);  PD 11 (44); CKD 24 (66.7) |  |  | HD 35 (15.5–60) m  PD 8 (5–32) m | MoCA | 7 |
| Kurella Tamura, M.2016 | USA | CRIC | eGFR > 20 mL/min/1.73 m^2^ | 3883 | 57.7 ± 11.0 | 2130 (54.9) | 1875 (48.3) | 3344 (86.1) | 845 (21.8) |  | MMSE | 8 |
| Rodríguez-Angarita, C. E.2016 | Colombia | cross-sectional | CKD 3-4 | 251 | 76.4±7.9 | 168 (66.9) | 60 (23.9) | 207 (82.5) |  |  | Short Neuropsychological Instrument in Spanish | 7 |
| Shea, Y. F.2016 | China | prospective | PD | 114 | 59±15.0 | 60 (53) | 68 (59.6) | 106 (93) |  |  | MoCA | 7 |
| McAdams-DeMarco, M. A.2015 | USA | cohort | HD | 324 | 54.8±13.3 | 183 (56.50) | 182 (56.2) | 324 (100) | 148 (45.7) |  | MMSE | 7 |
| Liu, G. L.2015 | China | cross-sectional | PD | 273 | 53.58±14.06 | 136 (49.81) | 73 (26.74) |  | 69 (25.27) | 26.80 (10.85–55.38) m | MMSE | 7 |
| Xu, R.2015 | China | cross-sectional | PD | 476 | 51.9±14.3 | 244 (51.4) | 113 (23.7) |  | 95 (20.1) | 26.3 (12.2–49.9) m | MMSE | 7 |
| Egbi, O. G.2015 | Nigeria | cross-sectional | CKD non-dialysis | 190 | 46.7 ± 13.4 | 113 (61.6) |  |  |  |  | six-item cognitive impairment test | 7 |
| Antunes, J. P.2015 | Portugal |  | CKD 2-5 | 246 | CKD 2 72.6±5.2  CKD 3-5 75.7±5.6 |  | 32 (13) | 109 (44) |  |  | MMSE | 7 |
| Seidel, U. K.2014 | Germany | prospective | CKD 3-5 | 119 | 61.5±15.7 | 75 (63.0) |  |  | 40 (34.2) |  | Wechsler memory scale | 7 |
| Jung, S.2013 | Korea |  | PD+HD | 56 | 54.2±10.2 | 27 (48.2) | 21 (37.5) |  | 22 (39.3) | 4.2±3.8 y | Korean version of the MMSE | 8 |
| Williams, U. E.2013 | Nigeria | case-control | CKD 3-5 | 79 | 39.7±11 | 52 (65.8) |  |  |  |  | Community Screening Interview for dementia | 7 |
| Yaffe, K.2013 | USA | CRIC | CKD | 588 | 65.3±5.6 | 309 (52.6) | 275 (46.8) |  | 168 (28.6) |  | MMSE | 7 |
| Sarnak, M. J.2013 | USA | cross-sectional | HD | 314 | 63+16 | 168 (53.50) | 150 (47.8) | 280 (89.2) | 138 (43.9) | 14 (7–35) m | MMSE | 7 |
| Post, J. B.2012 | USA | cross-sectional | HD | 50 | 63±10 | 26 (52) | 46 (92) |  |  | 2.5±3.8 y | MMSE | 7 |
| Kurella Tamura, M.2010 | USA | cross-sectional | HD | 383 | 51.6±13.3 | 238 (62) | 156 (42) |  |  | 2.6 (1.0-6.2) y | MMSE | 7 |
| Post, J. B.2010 | USA | cross-sectional | CKD+HD | 51 | CKD 72±12 HD 63±11 | 30 (58.9) | 48 (94.1) | 24 (47.1) |  | 2.2±2.3 y | MMSE | 7 |

KT, kidney transplantation; DM, diabetes mellitus; HT, hypertension; CVD, cardiovascular disease; CI, cognitive impairment; CKD, chronic kidney disease; HD, hemodialysis; PD, peritoneal dialysis; eGFR, estimated glomerular filtration rate; MoCA, Montreal Cognitive Assessment; MMSE, mini-mental state examination; DSST, Digit Symbol Substitution Test; CRIC, Chronic Renal Insufficiency Cohort; CORETH; BRINK, Brain in Kidney Disease; NICOLA, Northern Ireland Cohort for the Longitudinal Study of Ageing; SPRINT, Systolic Blood Pressure Intervention Trial; NHANES, National Health and Nutrition Examination Survey; ACE III, Adden brooke’s Cognitive Examination III.
