# Supplementary material for: Prevalence of cognitive impairment and its predictors among chronic kidney disease patients: A systematic review and meta-analysis
Source: PLoS One. 2024 Jun 3;19(6):e0304762. doi: 10.1371/journal.pone.0304762 (PMC11146742; doi:10.1371/journal.pone.0304762)
Supplement: S1 File — (DOCX) [file pone.0304762.s003.docx]

**S1 Fig** The pooled prevalence of cognitive impairment in CKD based on country

1. Africa


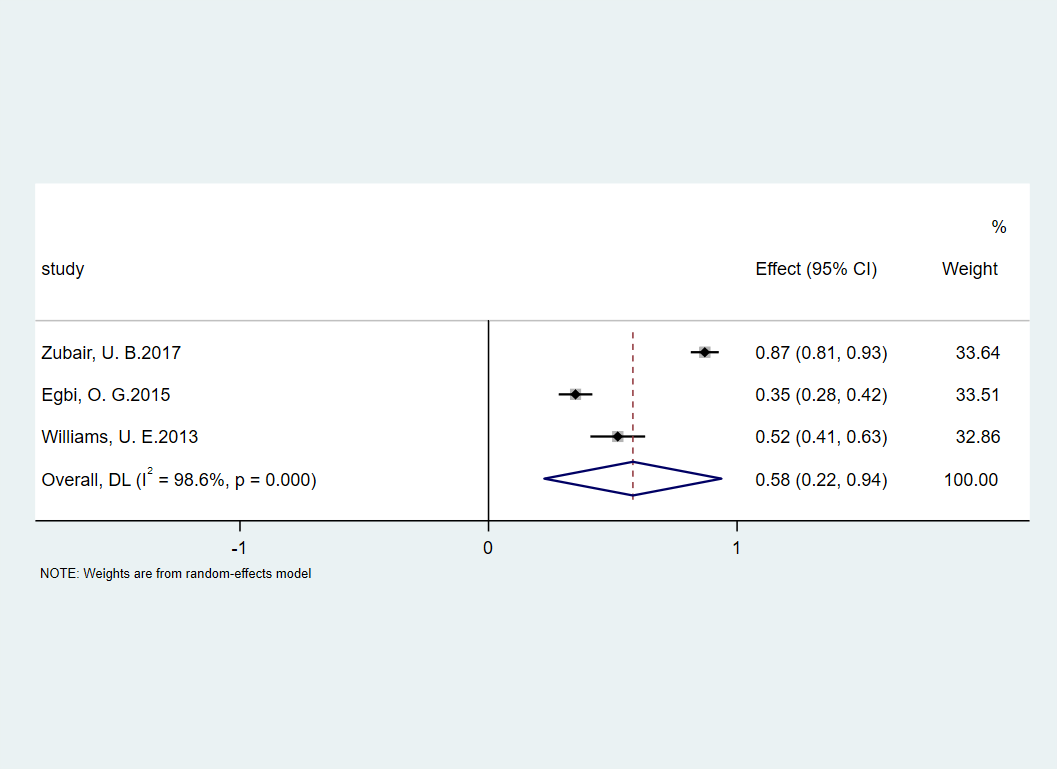


B. Australia


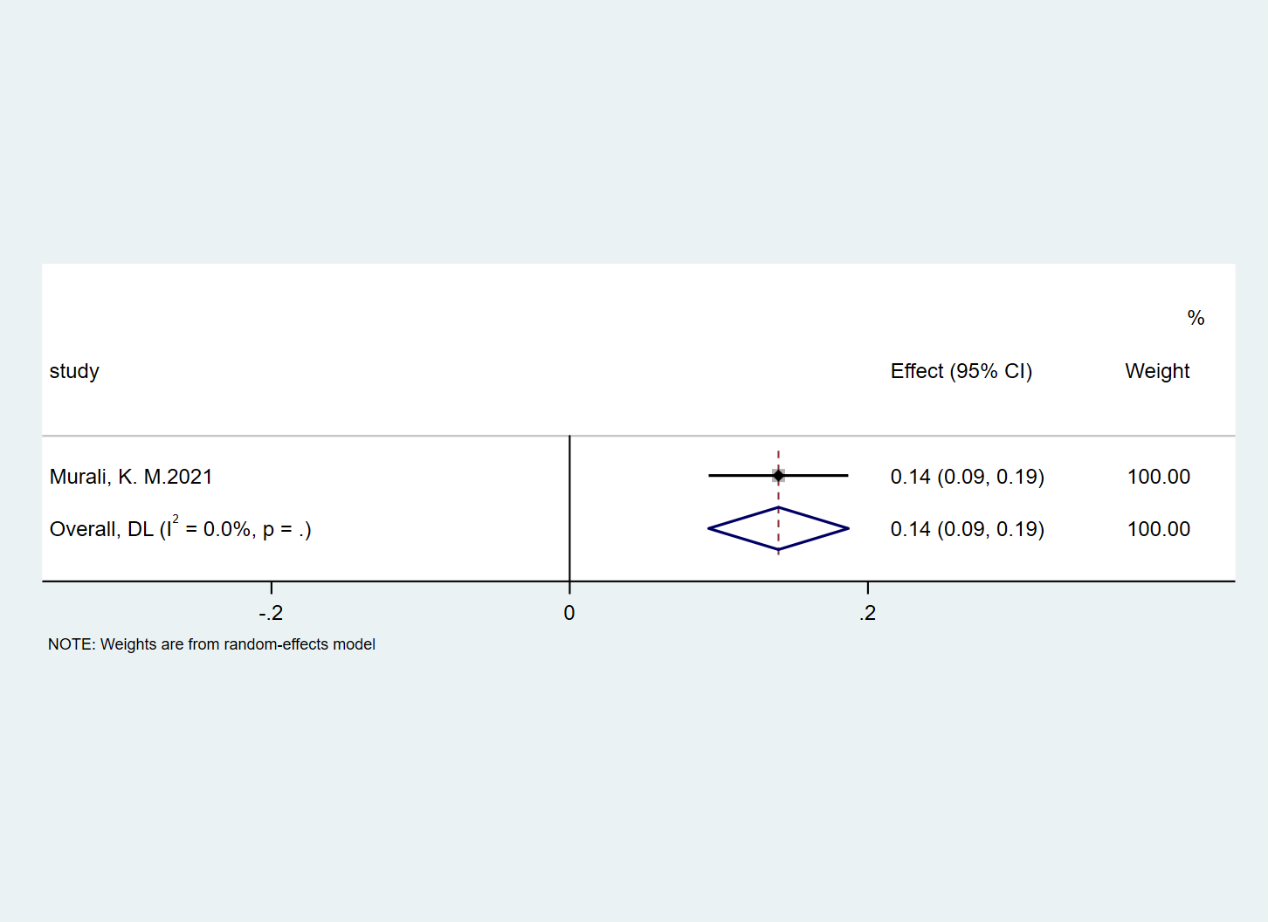


C. Asia


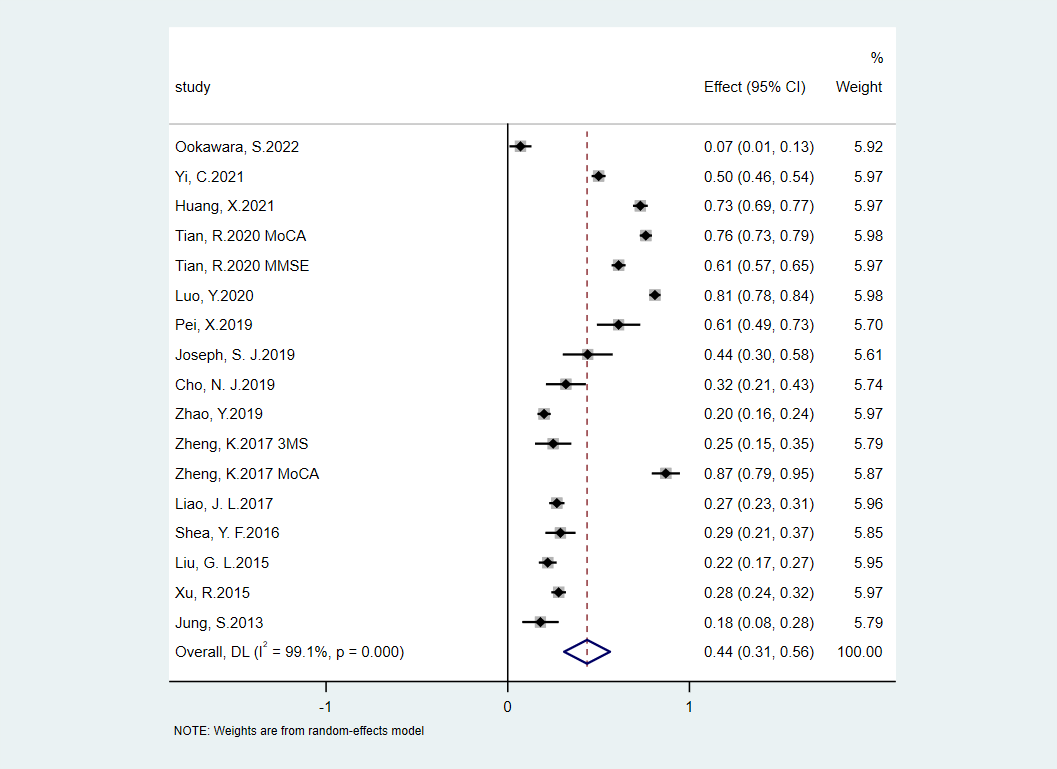


D. Europe


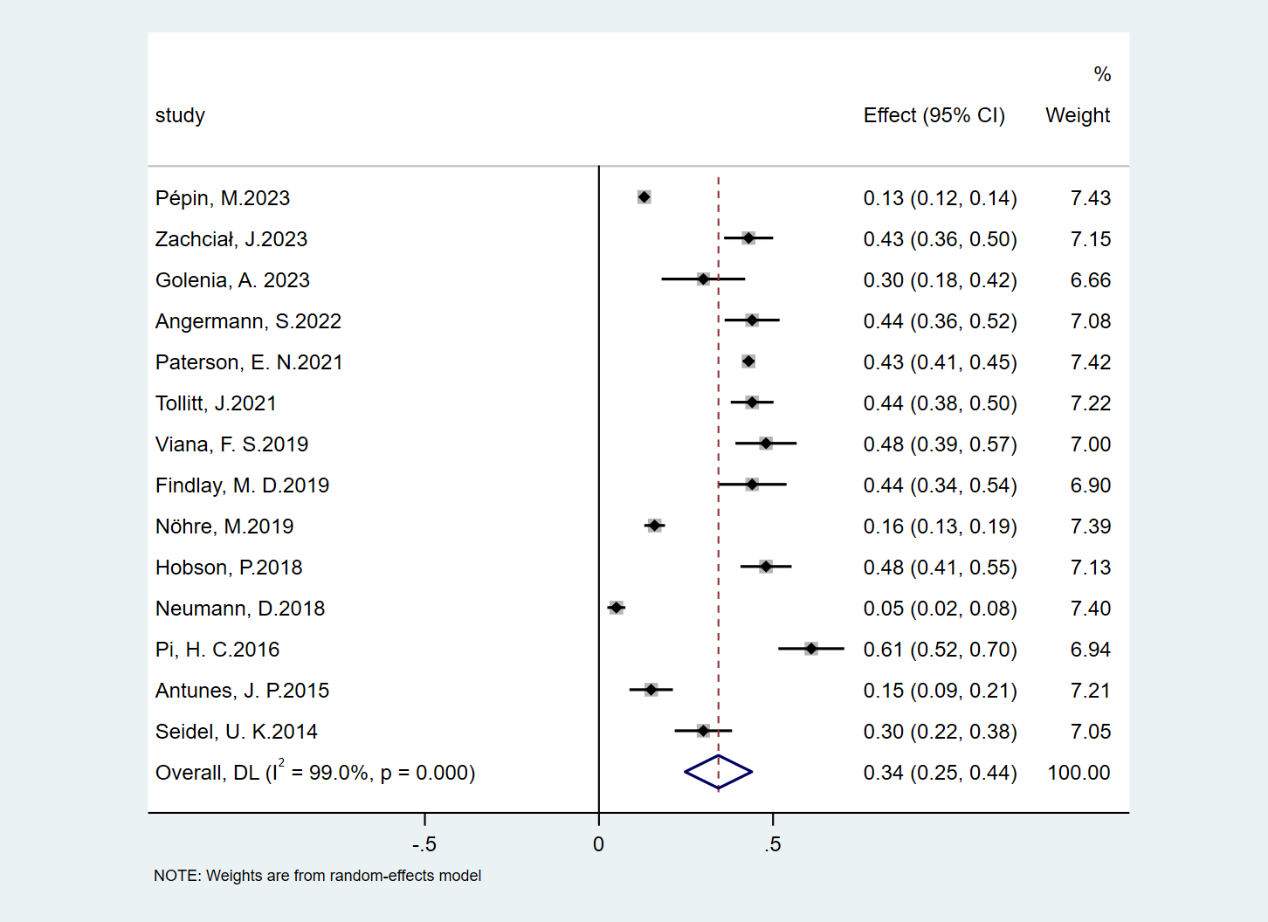


E. America


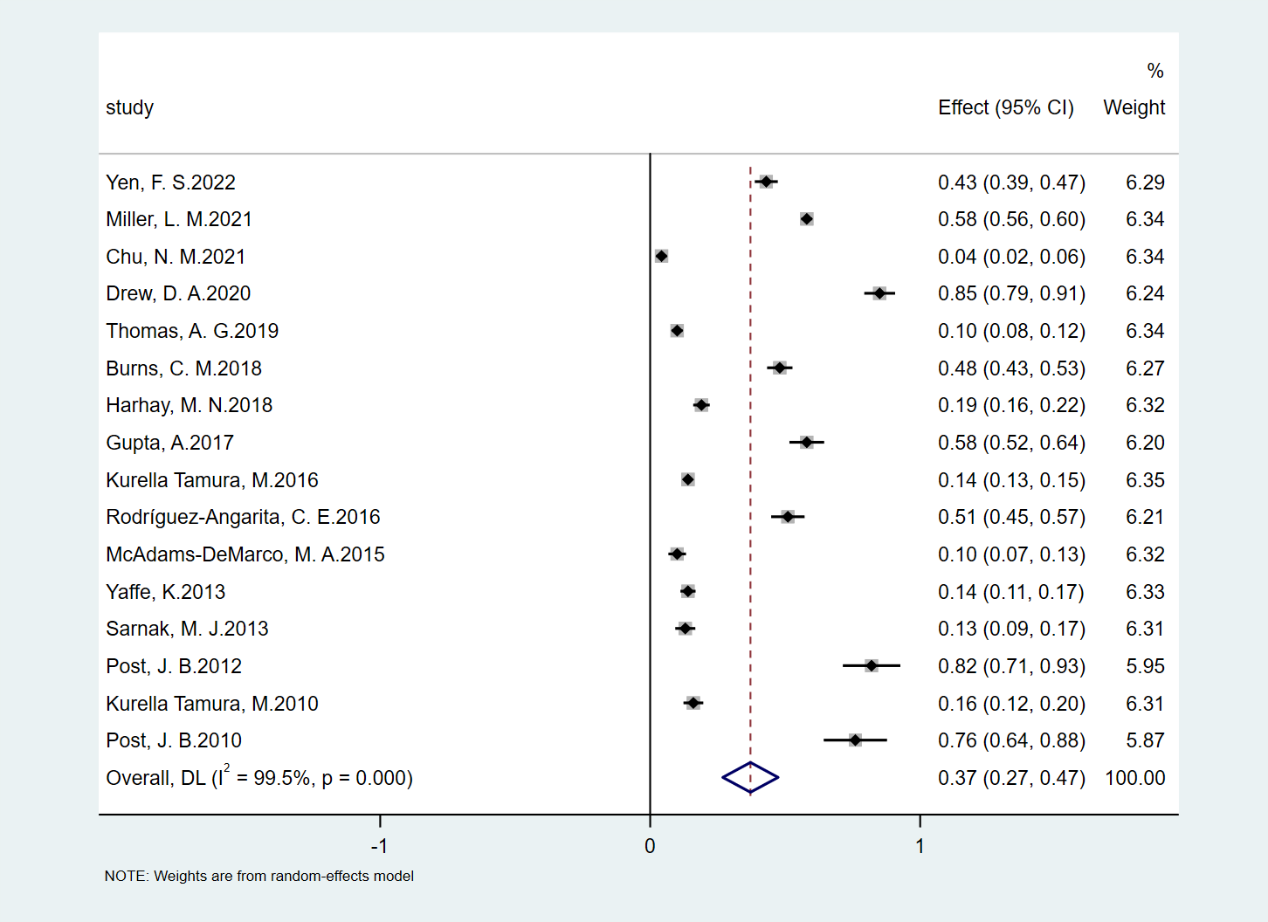


**S2 Fig** The pooled prevalence of cognitive impairment in CKD based on patients type


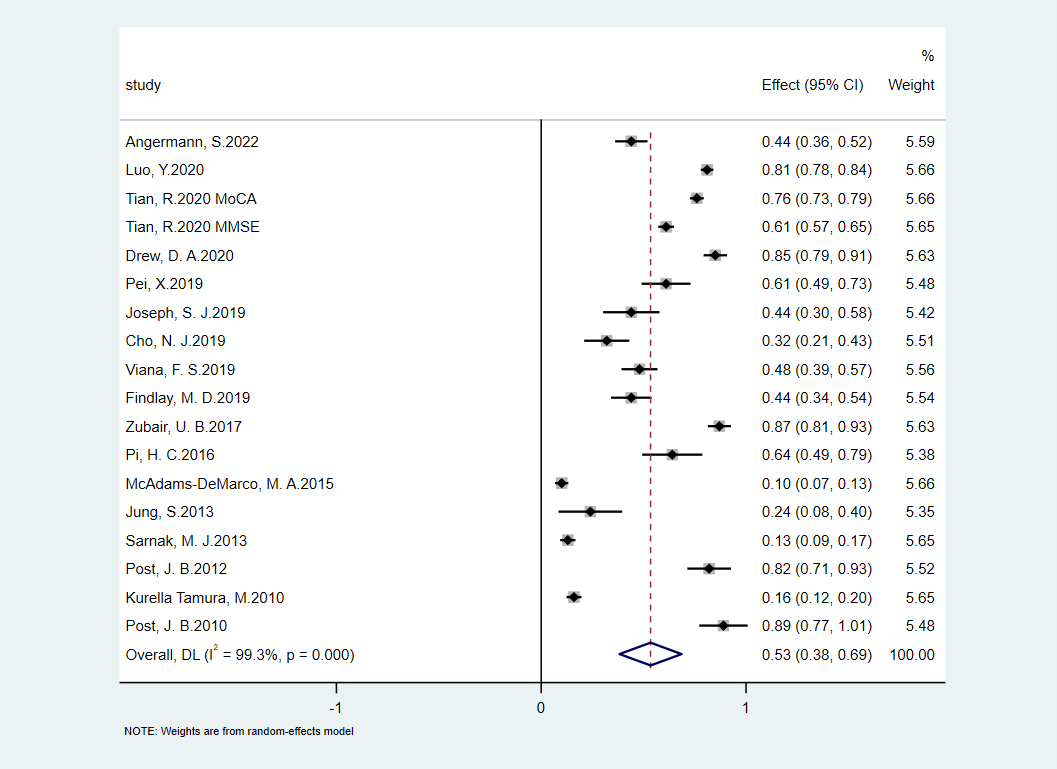
A. Hemodialysis


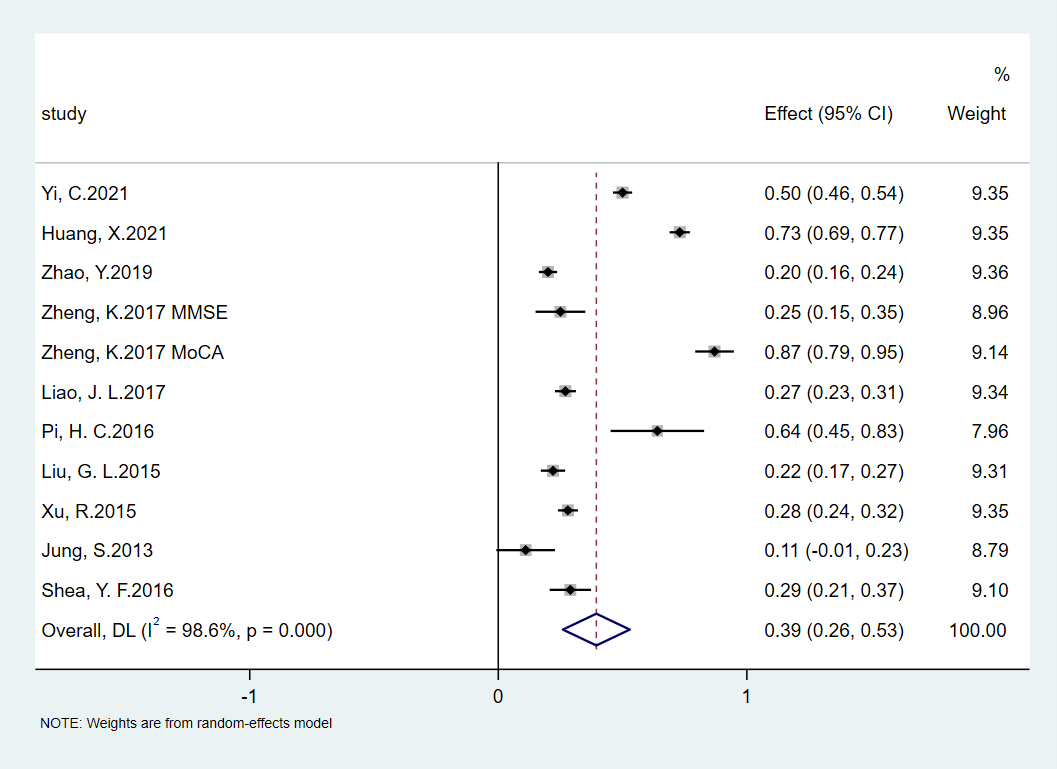
B. Peritoneal dialysis

C. Non-dialysis


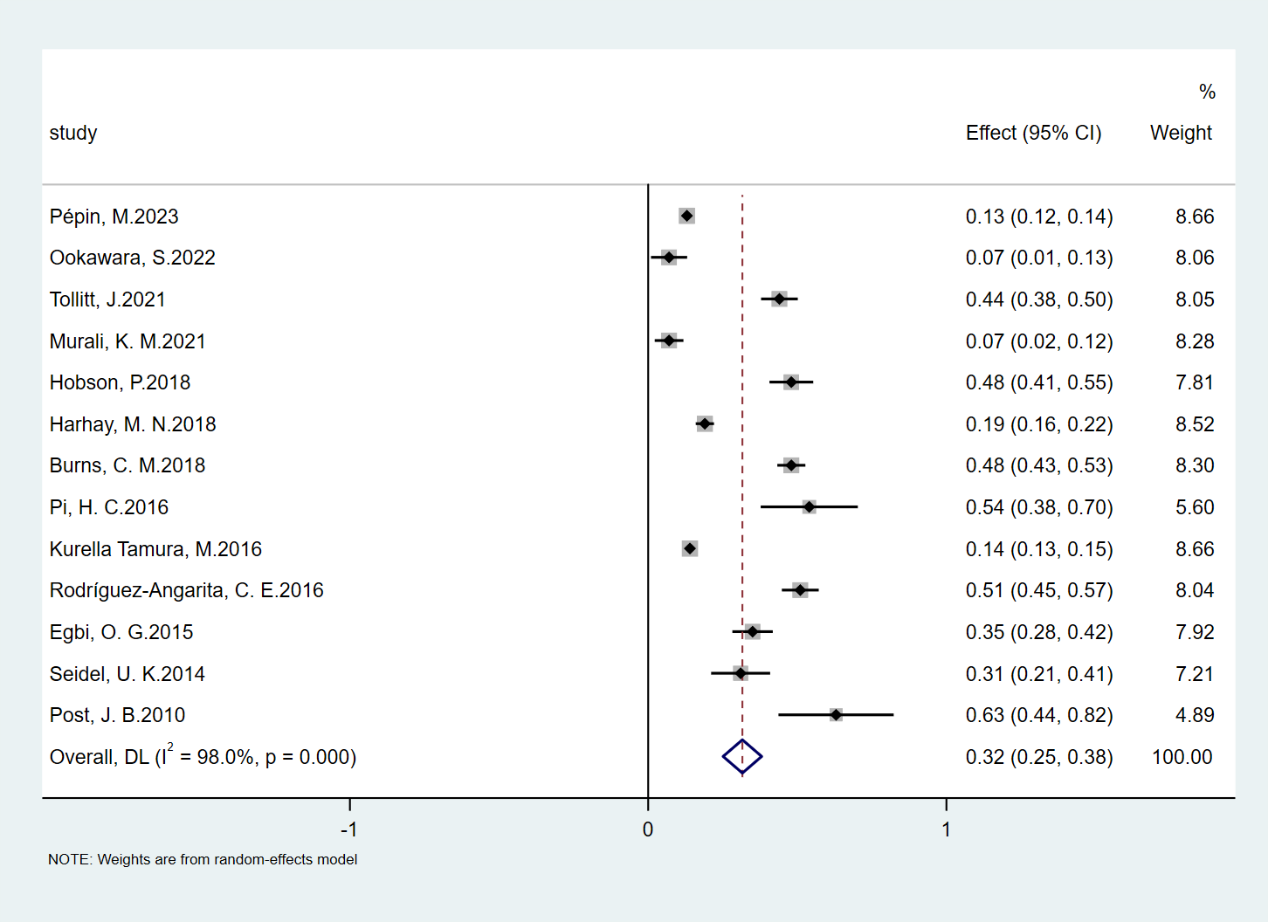


D. Kidney transplant


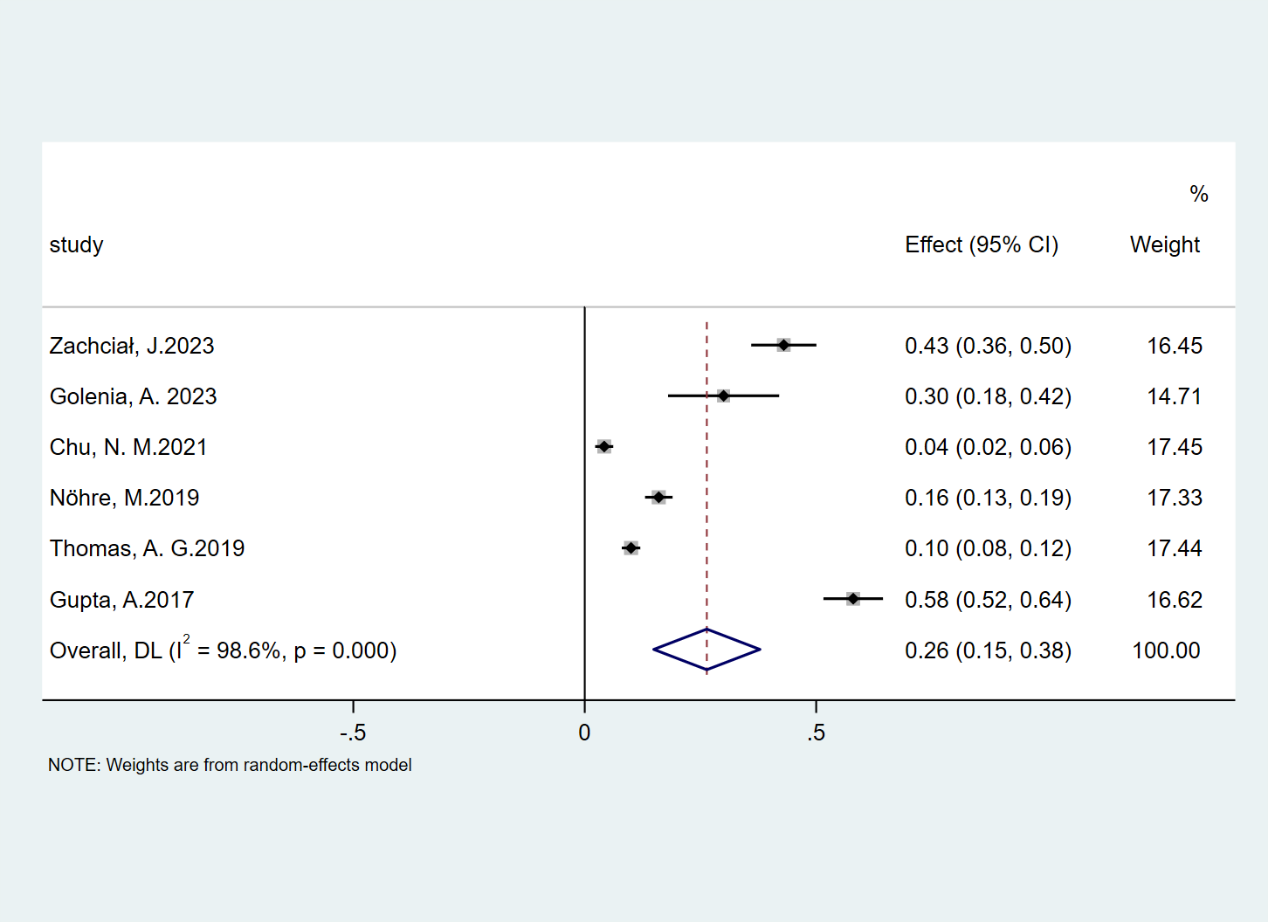


**S3 Fig** The pooled prevalence of cognitive impairment in CKD based on assessment tools

A. MoCA


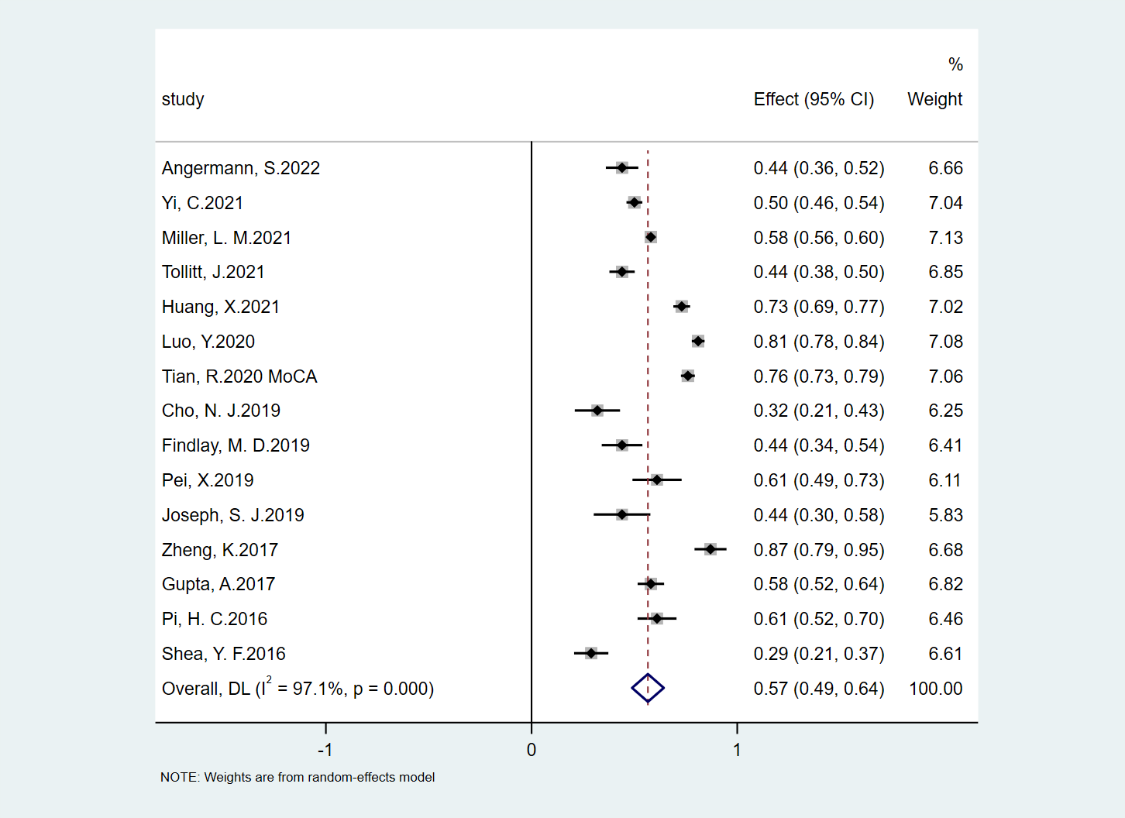


B. MMSE


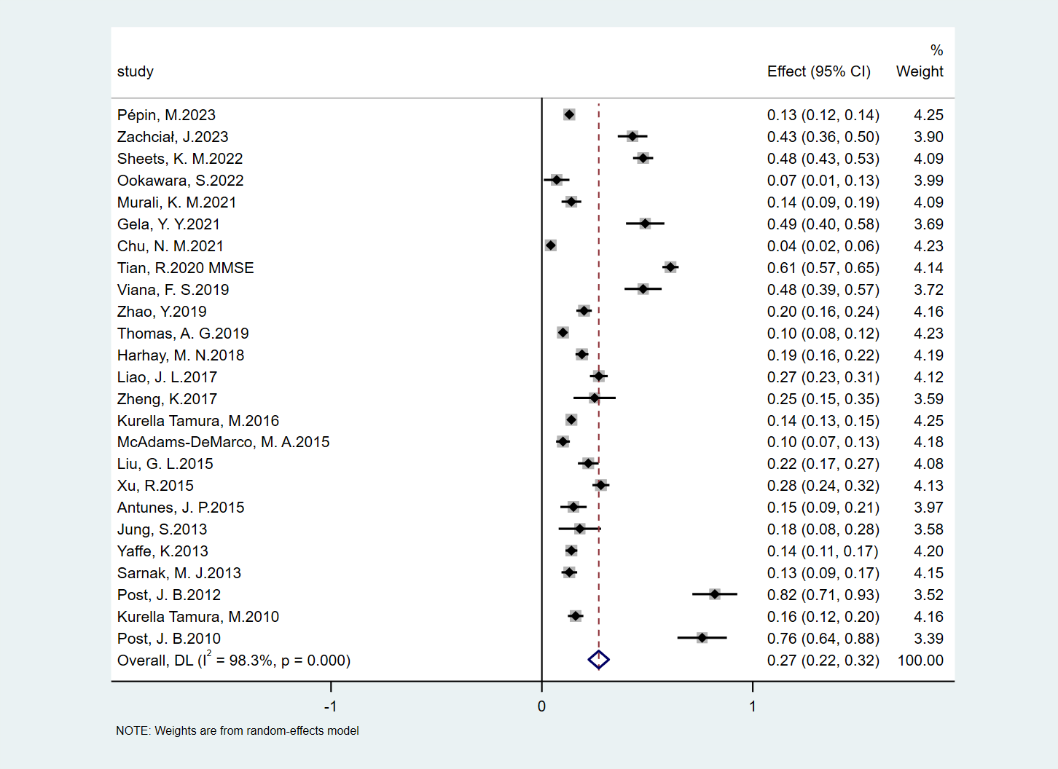


C. Addenbrooke’s Cognitive Examination III


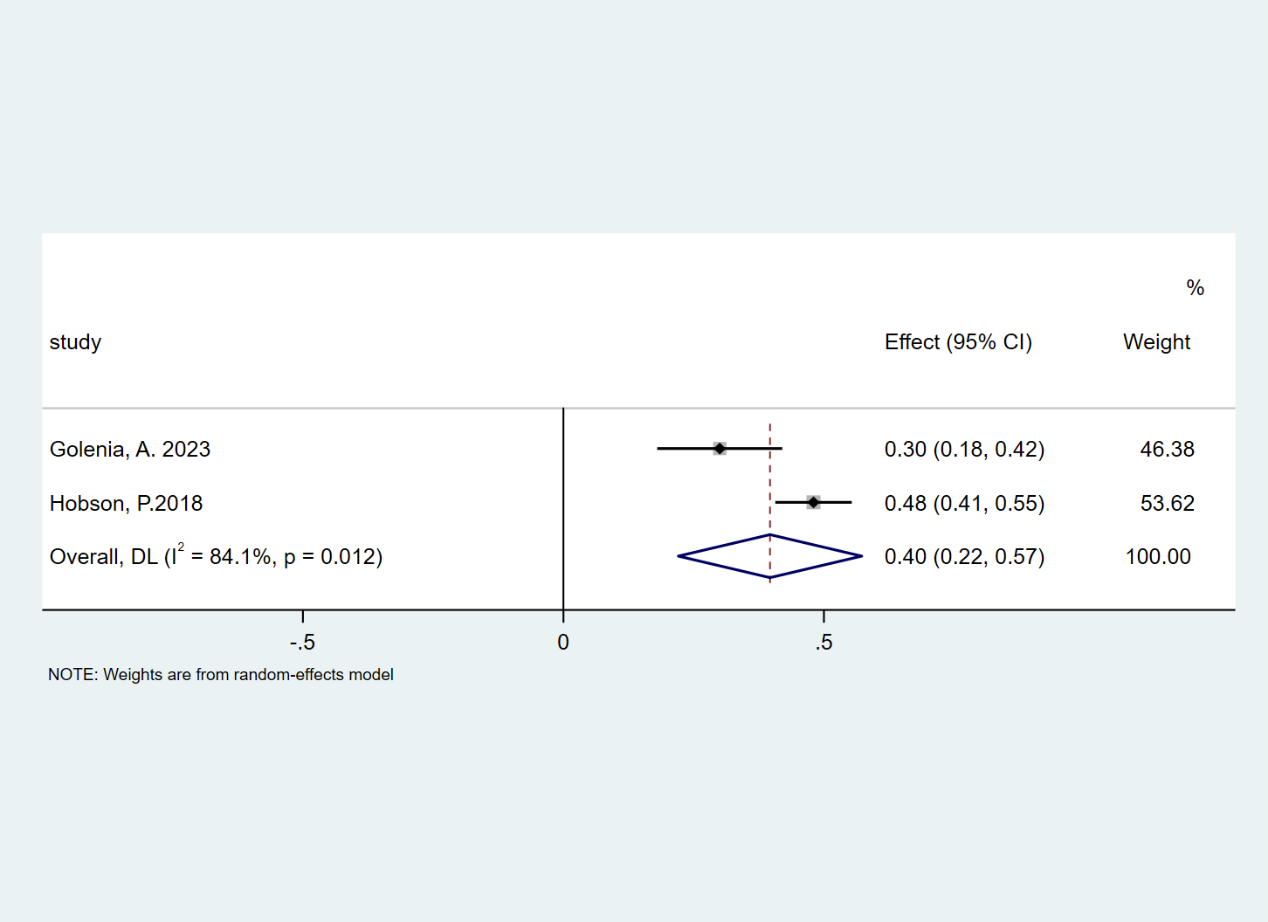


**S4 Fig** The pooled prevalence of cognitive impairment in CKD based on subdomains of cognition


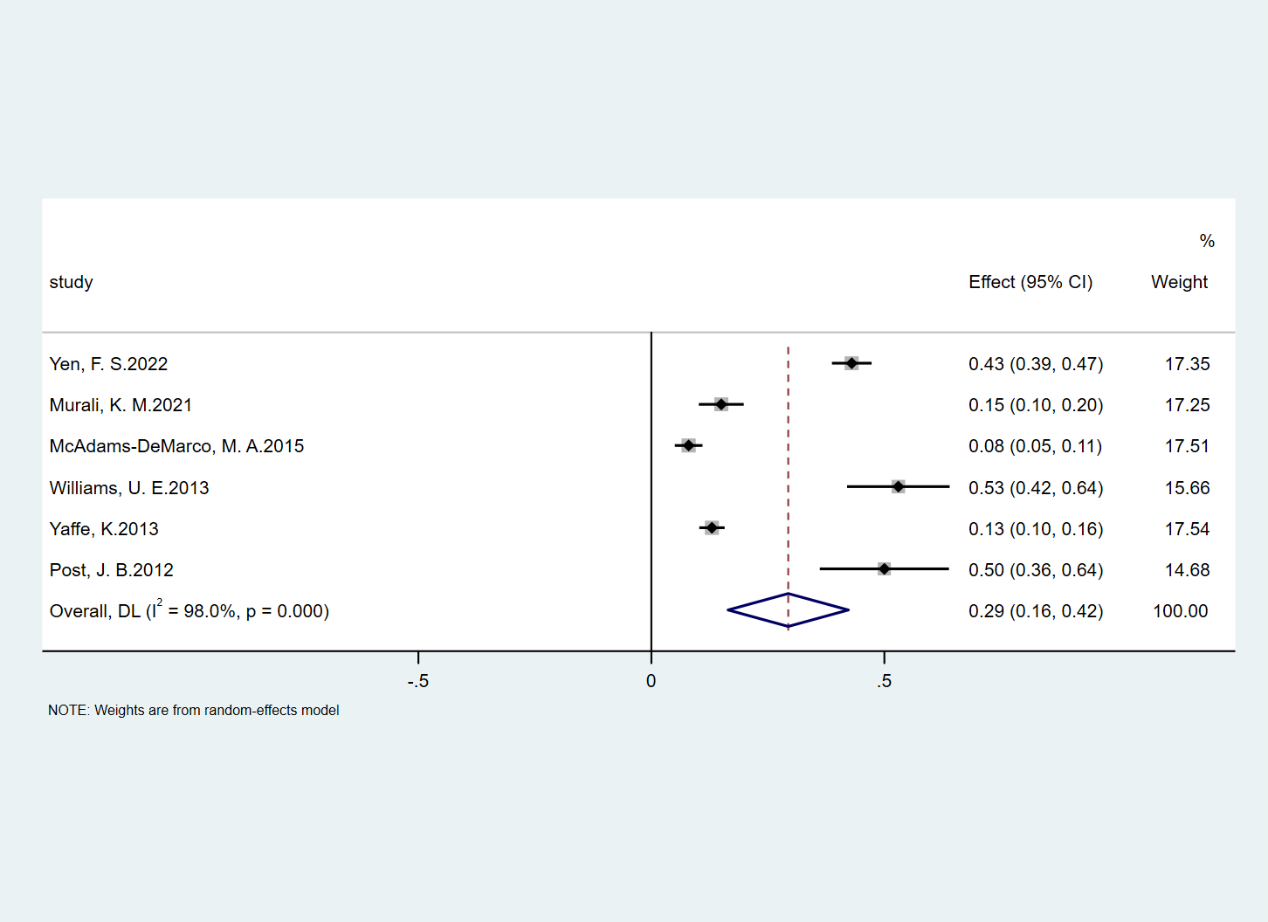
A. Attention

B. Executive function


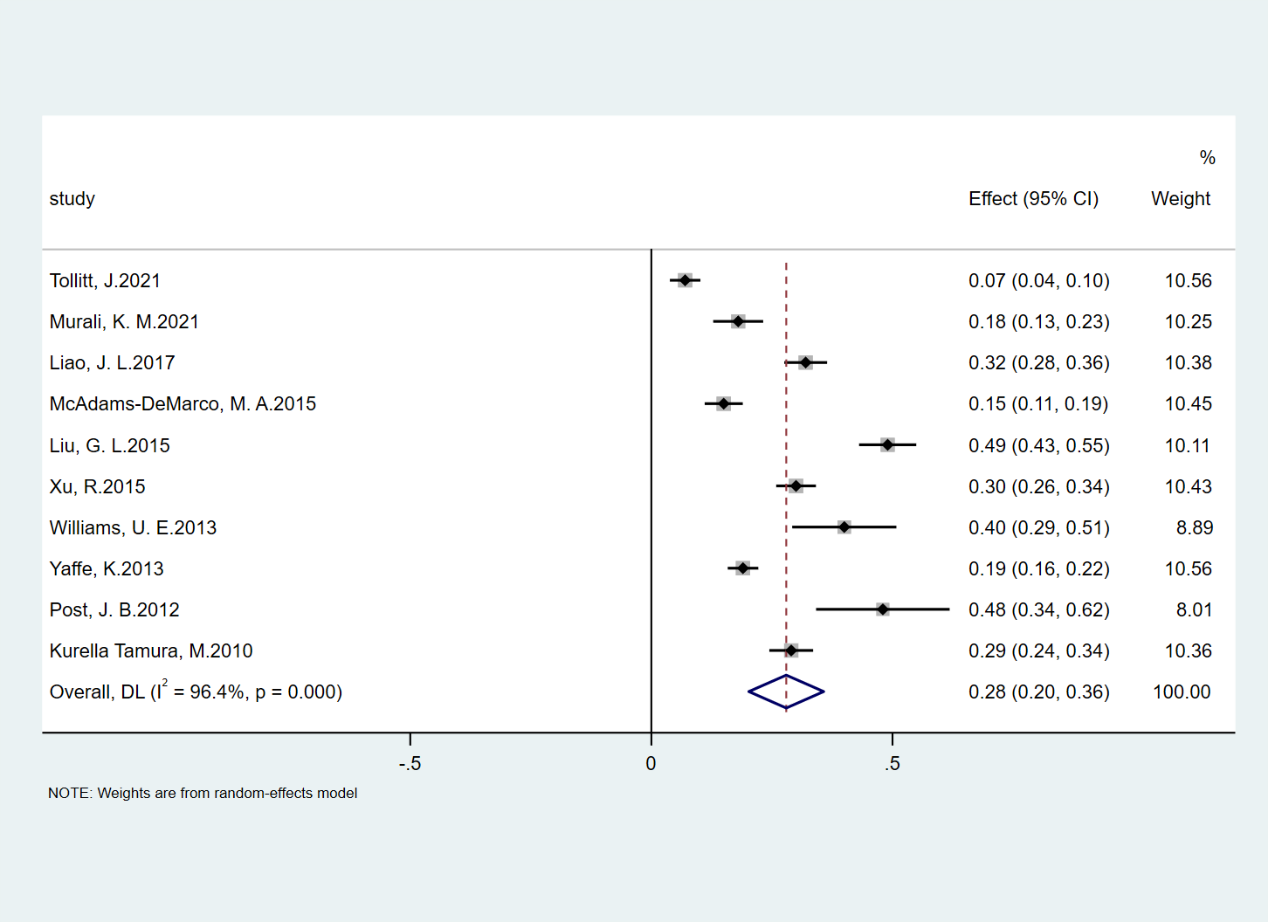


C. Language


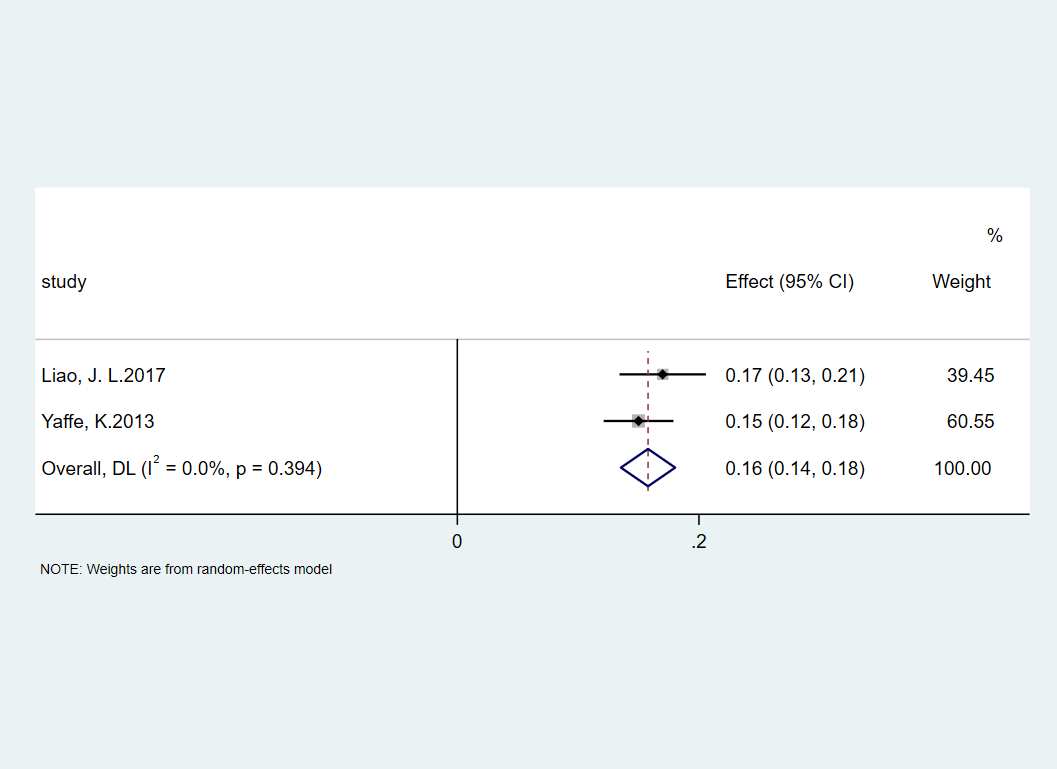


D. Memory


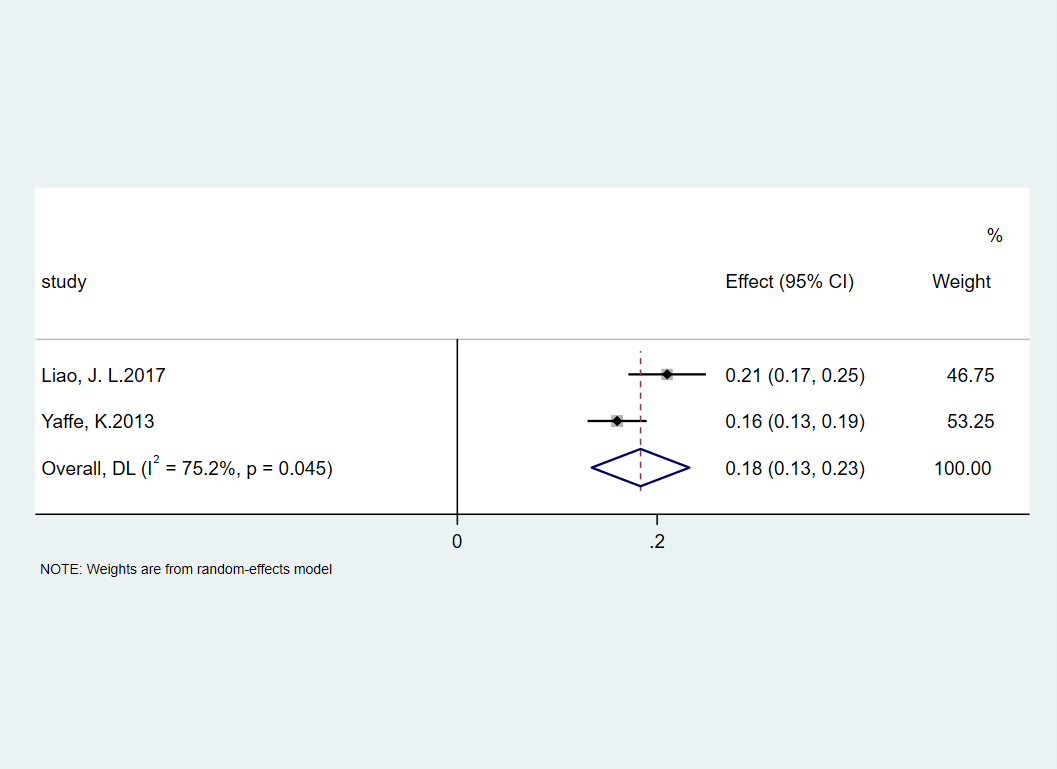


**S5 Fig** The pooled prevalence of cognitive impairment in CKD based on degree of cognitive impairment

A. Mild


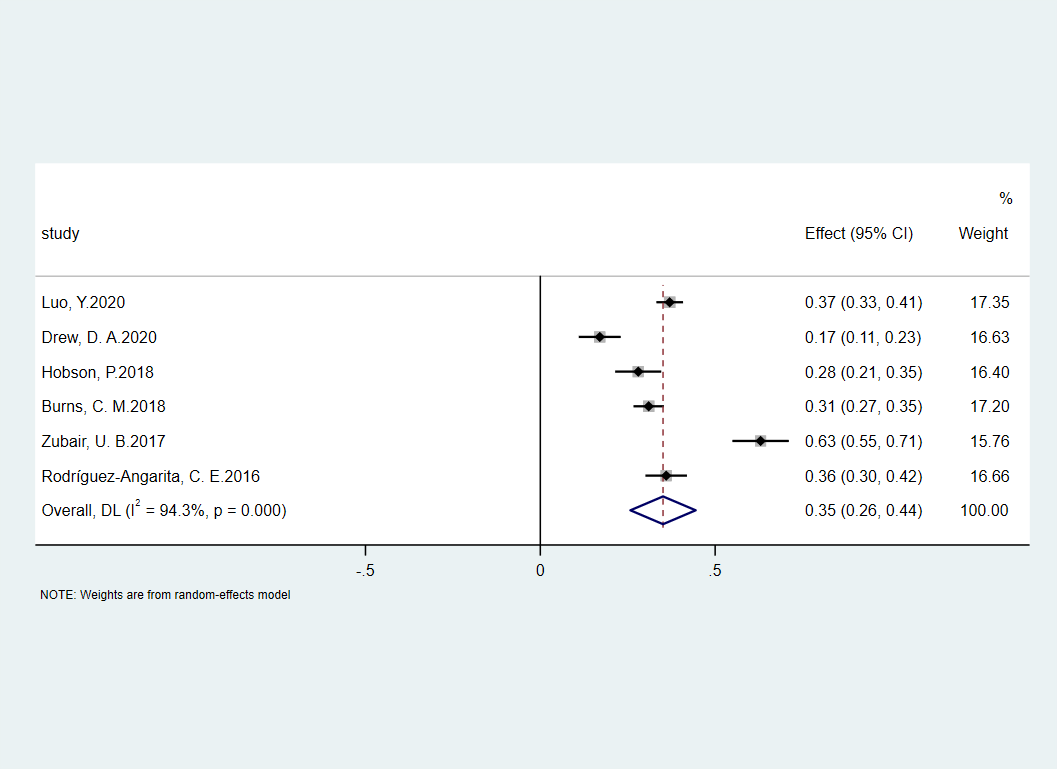


B. Major


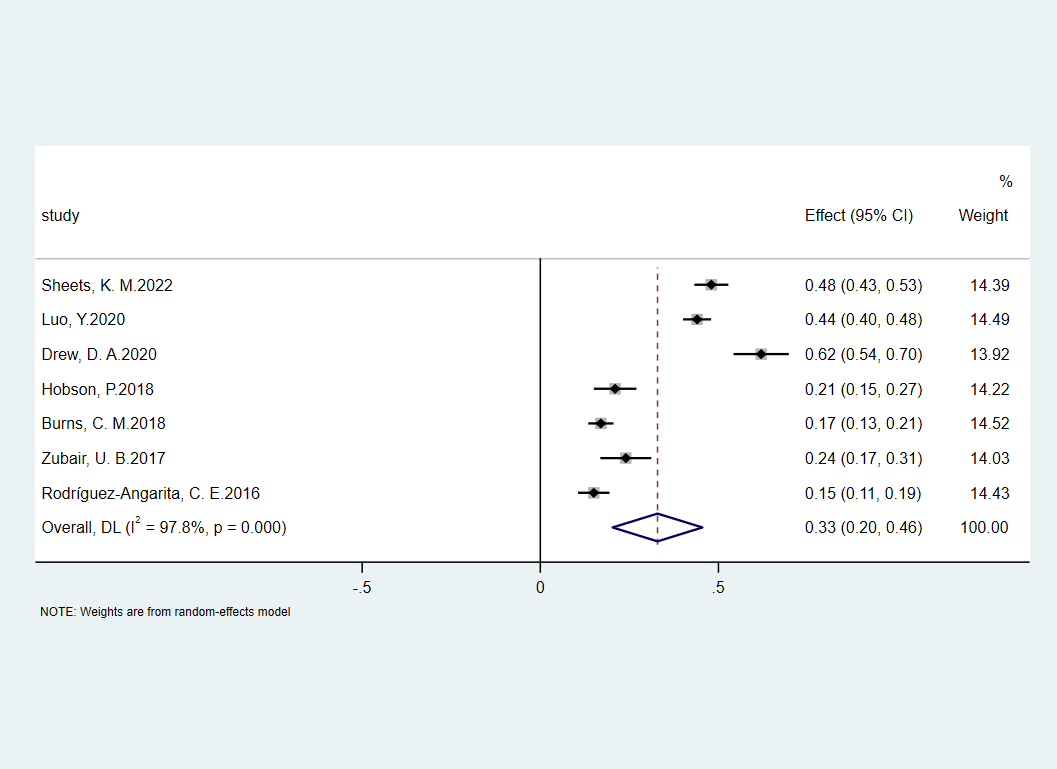


**S6** **Fig** The pooled prevalence of cognitive impairment in CKD based on study year


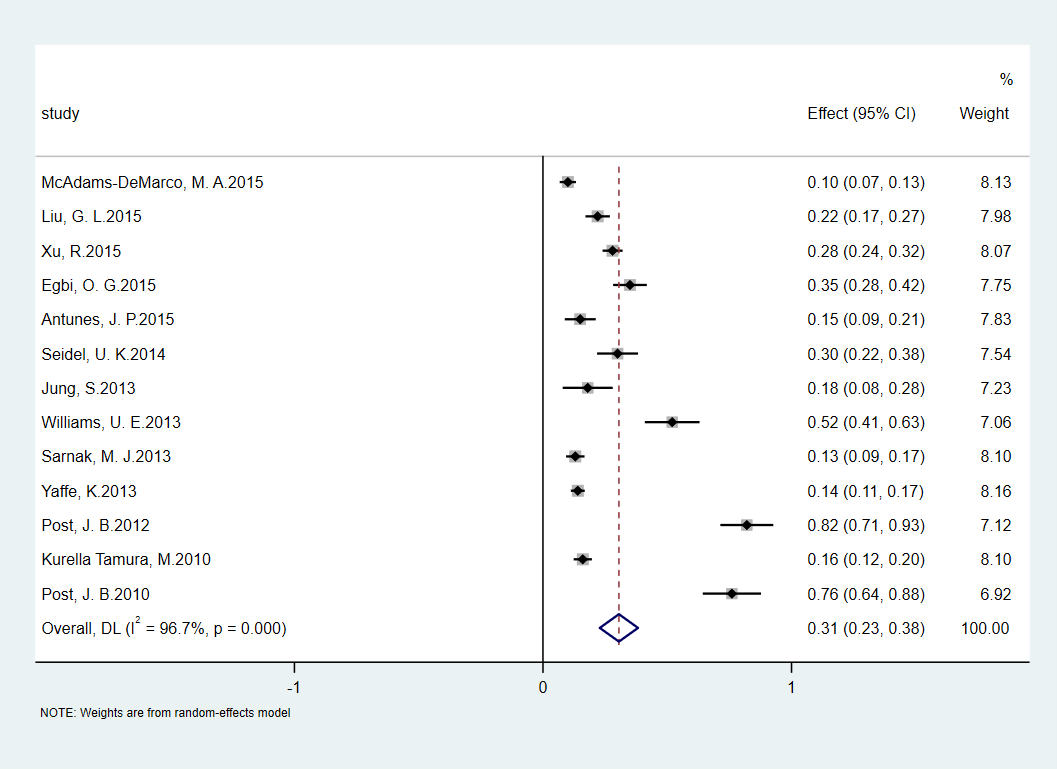
A. Year below 2015

B. Year over 2015


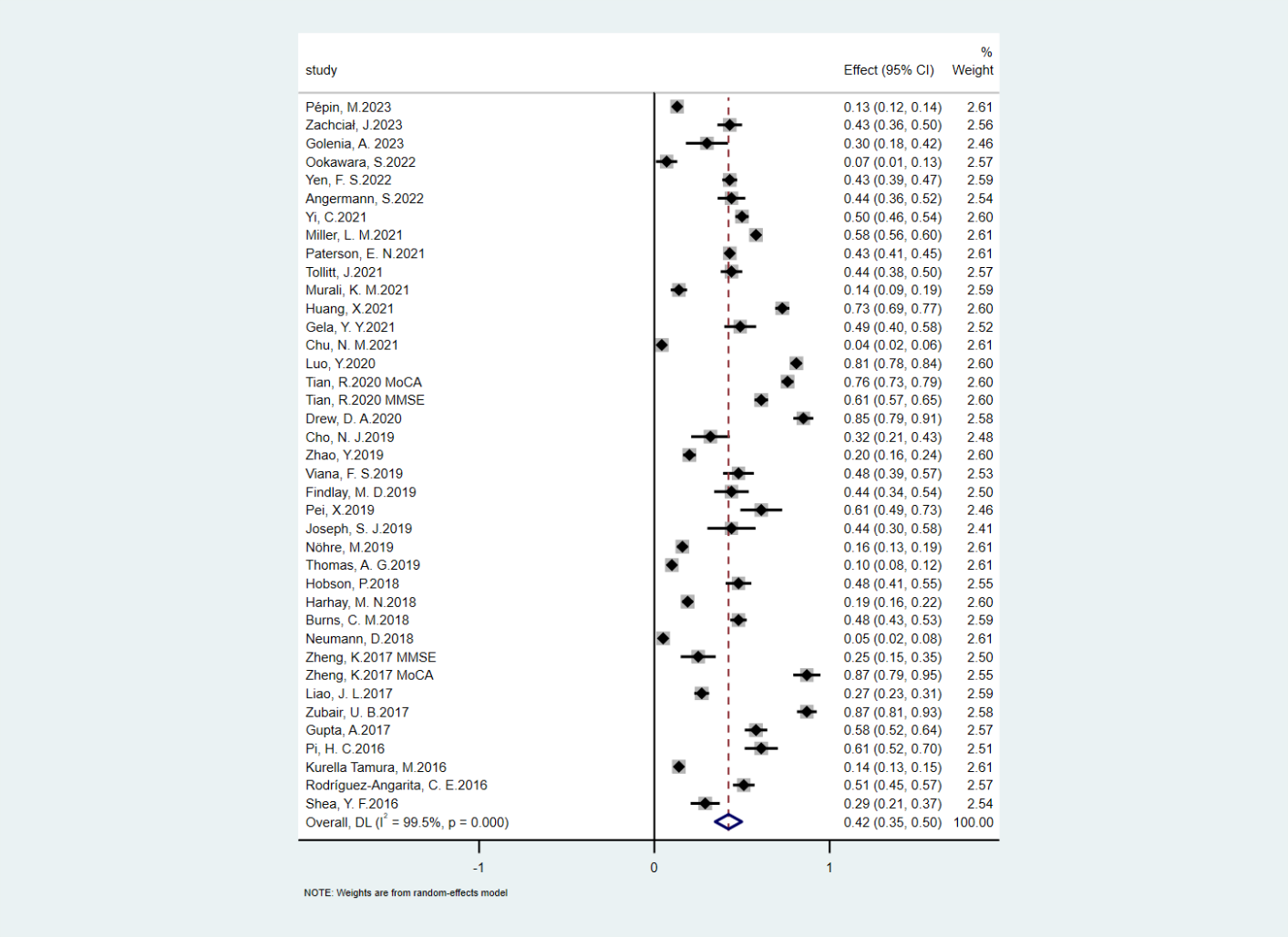


**S7 Fig** Associated factors with cognitive impairment among CKD patients


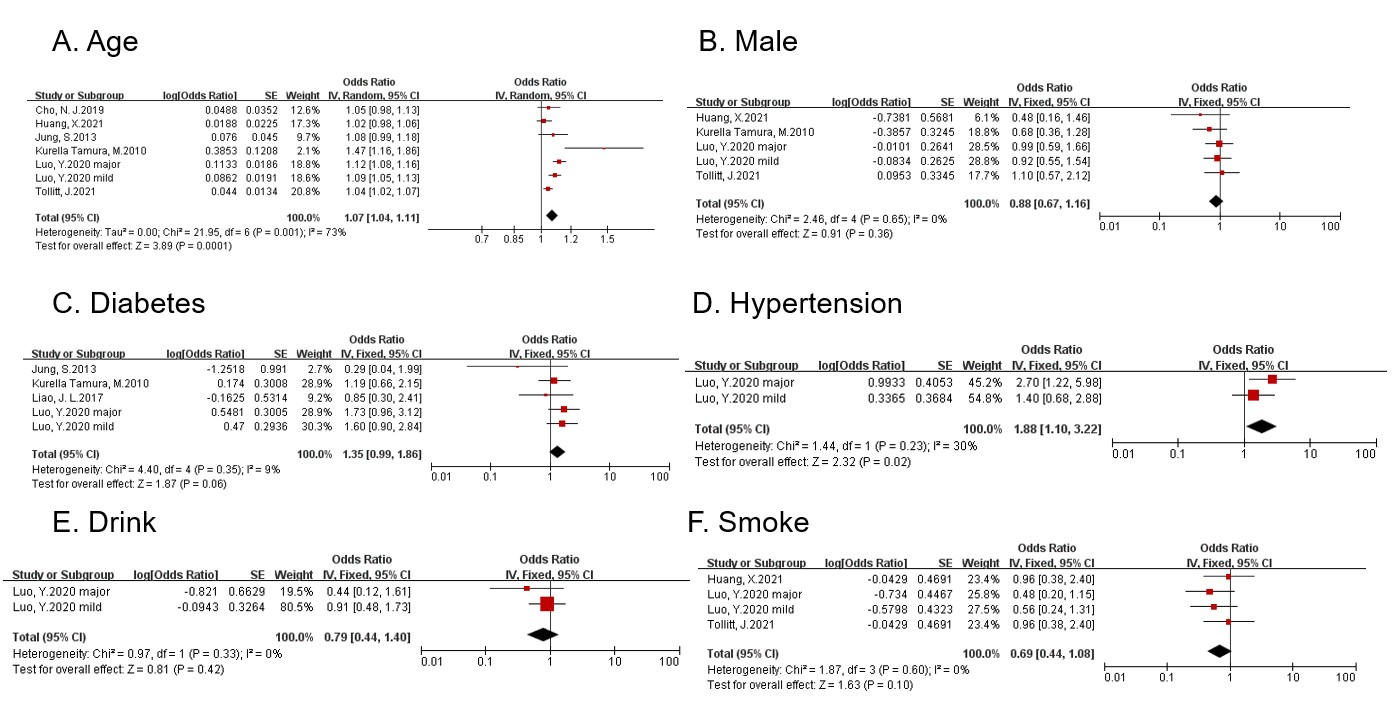


Search strategy for PubMed

(("Renal Insufficiency, Chronic") OR (chronic kidney disease) OR (hemodialysis) OR (peritoneal dialysis)) OR (pre-dialysis) AND (("Cognitive Dysfunction") OR (cognitive impairment))

Search strategy for Embase

#1 'chronic kidney failure':ti,ab,kw OR 'chronic kidney insufficiency':ti,ab,kw OR 'chronic renal failure':ti,ab,kw

#2 'cognitive defect':ti,ab,kw OR 'cognitive impairment':ti,ab,kw OR 'cognitive dysfunction':ti,ab,kw

#3 predialysis OR 'pre dialysis' OR dialysis OR haemodialysis.tw

#4 #1 OR #3

#5 #2 AND #4

Search strategy for Web of Science

#1 TS=("chronic kidney disease" OR "chronic kidney insufficiency" OR "chronic renal disease" OR "chronic kidney failure")

#2 TS=(hemodialysis OR peritoneal dialysis OR renal replacement therapy OR renal transplant OR renal replacement$ OR kidney replacement)

#3 TS=(cognitive impairment OR cognitive dysfunction)

#4 #1 OR #2

#5 #4 AND #3
